# Longitudinal multispectral image dataset for ToBRFV disease detection in tomato and pepper plants

**DOI:** 10.1016/j.dib.2026.112927

**Published:** 2026-06-04

**Authors:** Rifat Edizkan, Feyza Yılmaz, Pelin Keleş Öztürk, Şefika Yavuz, Ömer Nezih Gerek, Hasan Serhan Yavuz, Serkan Önder, Davut Keleş

**Affiliations:** a Department of Electrical and Electronics Engineering, Faculty of Engineering and Architecture, Eskisehir Osmangazi University, Eskisehir 26480, Türkiye; b Biological Control Research Institution, Adana 01321, Türkiye; c Department of Electrical and Electronics Engineering, Faculty of Engineering, Eskisehir Technical University, Eskisehir 26555, Türkiye; d Department of Plant Protection, Faculty of Agriculture, Eskisehir Osmangazi University, Eskisehir 26160, Türkiye; e Department of Horticulture Research, Republic of Turkey Ministry of Agriculture and Forestry General Directorate of Agricultural Research and Policies, Ankara 06800, Türkiye

**Keywords:** Tomato brown rugose fruit virus (ToBRFV), Multispectral imaging, Computer vision, Early disease detection, Tomato plants, Pepper plants, Agricultural engineering

## Abstract

ToBRFV is a major threat to tomato and pepper crops because it spreads quickly and survives for a long time in the environment. Since there are few ways to control it after infection, early detection before symptoms are visible is crucial. Yet, only limited public datasets are available for this research. We present one of the first openly accessible, longitudinal multispectral image dataset dedicated to ToBRFV detection. In this study, two tomato cultivars and two pepper cultivars, all of which are commercially important and widely cultivated in greenhouses, were selected. Using these plants ensures that the dataset reflects real-world agricultural practices and captures variability across commercially grown types. Both healthy and ToBRFV-inoculated plants from each cultivar were included in the imaging process. All plants were cultivated under fully controlled greenhouse conditions in Adana Province, Türkiye. Healthy and infected tomato plants were grown in two separate greenhouses to prevent cross-contamination. Imaging was conducted over a 29-day period using Red-Green-Blue (RGB) and Visible Near Infrared (VNIR) cameras, including narrowband captures at 800 nm and 1000 nm, from multiple viewing angles. Infection status was confirmed via Reverse Transcription quantitative Polymerase Chain Reaction (RT-qPCR) analysis at multiple time points. The dataset is organized into four clean, labelled subsets and released under a CC BY 4.0 license. This resource provides unique opportunities for developing and benchmarking computer vision and machine learning approaches for pre-symptomatic plant disease detection, spectral feature analysis, and integration into precision agriculture systems. By combining controlled experimental design, spectral diversity, and open access, it establishes a robust foundation for cross-disciplinary research in plant pathology, agricultural engineering, and artificial intelligence.

Specifications Table

**Table:** 

Subject	Engineering & Materials science
Specific subject area	Pre-symptomatic detection of ToBRFV infection, AI-based detection of ToBRFV in tomato and pepper plants, Temporal and spectral analysis of ToBRFV disease
Type of data	TIFF: 72 dpi, 24-bit PNG: 72 dpi, 8-bit
Data collection	Tomato and pepper plants were cultivated in two fully controlled greenhouses in southern Turkey. A total of 167 tomato and 180 pepper plants, each grown in an individual pot, were used. Among these, 90 tomato and 90 pepper plants were mechanically inoculated with a confirmed ToBRFV isolate, and inoculation was repeated after one week. Separate greenhouses housed healthy and infected plants to prevent cross-contamination. Images were captured using a Nikon D90 Digital Single-Lens Reflex (DSLR) camera (Nikon AF-S DX Nikkor 18–55 mm f/3.5–5.6G VR) and an MT-MIMC-V6418 VNIR camera (f/0.95–25 mm) under natural lighting. The VNIR camera covers 400–1100 nm, with narrowband images at 800 nm and 1000 nm obtained using additional bandpass IR filters. Four images per plant were recorded, excluding blurred or overexposed shots. The camera angle was set to 0°, 5°, or 15° to capture ToBRFV symptoms from different perspectives. DSLR RAW images were converted to Tagged Image File Format (TIFF), while VNIR images were stored in Portable Network Graphics (PNG) format. All images were cropped to isolate the plant region while preserving their original resolution. The final dataset comprises four subsets: The final dataset comprises four subsets: RGB, full-range VNIR (400–1100 nm), VNIR at 800 nm, and VNIR at 1000 nm.
Data source location	Data were collected in two greenhouses at the Adana Biological Control Research Institution (37.00° N, 35.32° E), while all imaging and dataset preparation were conducted at Eskisehir Osmangazi University (39.77° N, 30.52° E).
Data accessibility	Repository name: ZENODO Data identification number: 10.5281/zenodo.17244968 Direct URL to data: https://doi.org/10.5281/zenodo.17244968
Related research article	None.

## 1 Value of the Data

• This dataset represents one of the first openly accessible, longitudinal multispectral image resources specifically developed for ToBRFV detection in commercially important tomato and pepper cultivars. It includes RGB and VNIR (400–1100 nm) imagery, as well as narrowband captures at 800 nm and 1000 nm.
• Images were collected over a 29-day period from healthy and RT-qPCR-confirmed infected plants grown under fully controlled greenhouse conditions, providing a reliable foundation for early-stage and pre-symptomatic disease detection research.
• Its multi-angle and multi-spectral structure support studies on modality fusion, spectral feature importance, temporal disease progression, and early physiological responses prior to visible symptoms.
• The dataset enables rigorous development, comparison, and benchmarking of computer vision and machine learning algorithms in precision agriculture applications.
• Freely available under a CC BY 4.0 license, it promotes reproducibility, open science, and collaboration across plant pathology, agricultural engineering, and AI research communities.

## 2 Background

ToBRFV is an emerging tobamovirus that infects tomato (Solanum lycopersicum) and pepper (Capsicum sp.) plants cultivated in both open fields and greenhouses, spreading rapidly under favorable conditions [1]. First identified in Israel and Jordan in 2014–2015, it has since been reported in China, Mexico, the United States, and several EPPO member countries [2]. In tomatoes, symptoms include mosaic, mottling, leaf curling, and fruit discoloration, whereas peppers exhibit mosaic, yellowing, and deformation of leaves and fruits [3,4]. Transmission occurs through seeds, infected plant debris, and human or mechanical contact [5]. Early detection is difficult because plants often remain asymptomatic during the incubation period, leading to severe yield losses. While some longitudinal ToBRFV datasets have been published [6,7], and broader plant disease collections such as PlantSeg and PlantDoc exist [8,9], none provide a comprehensive, multispectral, and temporally structured dataset dedicated to ToBRFV. The dataset introduced in this study, ToBRFV-LMID, addresses this gap by providing RGB and VNIR images of tomato and pepper plants under controlled greenhouse conditions. This dataset fills a critical gap in plant disease diagnostics and establishes a stronger foundation for AI-based models that generalize beyond a single plant type.

## 3 Data Description

The longitudinal multispectral image dataset developed in this study comprises 40,716 images collected from totally 167 tomato plants and 180 pepper plants. Among these, the virus-inoculated groups comprised 90 tomato plants (45 designated as Tomato I and 45 as Tomato II) and 90 pepper plants (45 designated as Pepper I and 45 as Pepper II), all of which were mechanically inoculated with a confirmed isolate of TOBRFV. The remaining 77 tomato plants and 90 pepper plants were planted as healthy controls. Imaging was conducted over a 29-day period, beginning on the day of inoculation, with captures taken at predefined daily intervals in the greenhouses of the Adana Biological Control Research Institution.

**Fig. 1:**
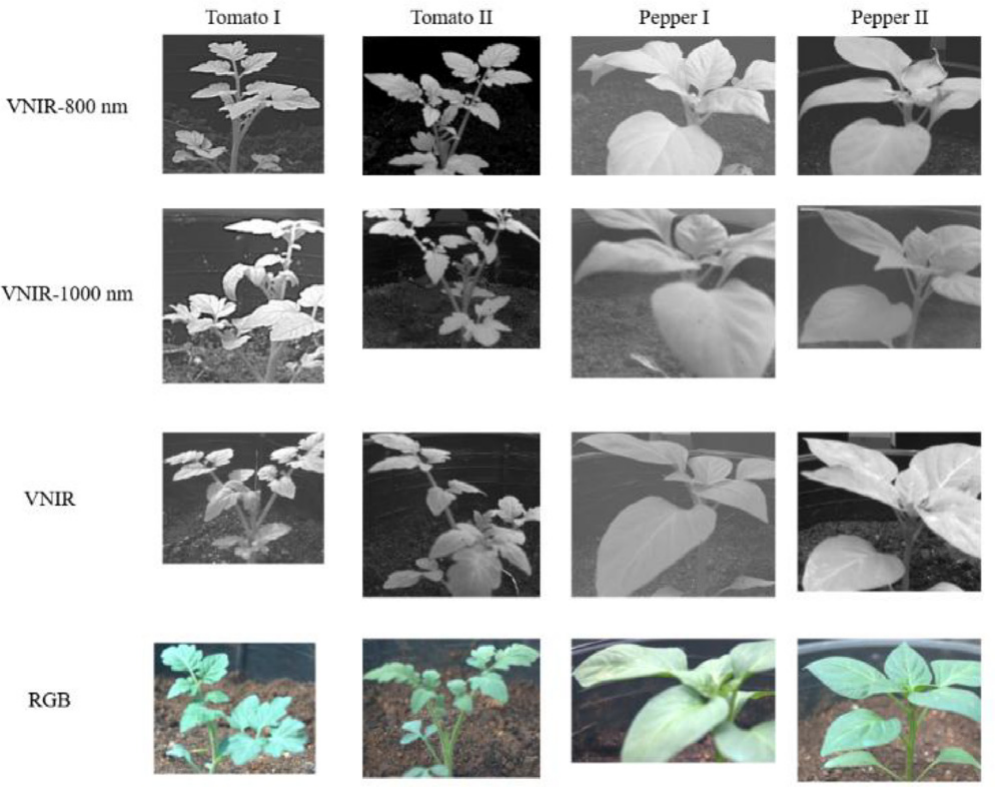
Sample cropped images of the four modalities.

Images were captured from three camera angles (0°, 5°, and 15° relative to the plant origin) to observe symptoms from different perspectives. The DSLR camera was configured to store photographs in NEF (Nikon Electronic Format) format to preserve maximum image quality. These files were converted to standard RGB TIFF format using the third party program DCRAW, which applies white balance based on metadata coefficients embedded in the NEF file [10]. The resulting TIFF images have a 24-bit depth. The VNIR camera stores images in PNG format with 8-bit depth. After cropping, the images retained their original resolution. Cropped sample images of the four modalities are shown in Fig. 1.

The dataset is organized into four main folders: RGB, full-range VNIR (400–1100 nm), VNIR at 800 nm, and VNIR at 1000 nm. Each main folder contains subfolders for each plant cultivar, and within each cultivar folder, images are further divided into healthy and virus-infected plants. The description of dataset is given in Table 1.

**Table 1:** Brief description of dataset folders.

Plant	Tomato and Pepper
Number of folders	4
Dataset folders	RGB, VNIR 400–1100 nm, VNIR 800, and VNIR 1000
File size	PNG: 2.72 GB TIFF: 9 GB
Dataset size	RGB: 9 GB VNIR 400–1100: 890 MB VNIR 800: 780 MB VNIR 1000: 1.05 GB

## 4 Experimental Design, Materials and Methods

**Fig. 2:**
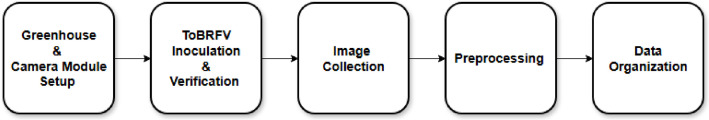
Workflow of dataset preparation process.

The experimental setup for carrying out multispectral imaging was designed in detail by collaborating multidisciplinary studies. In our study, the dataset development involved the following steps: greenhouse and camera module setup, ToBRFV inoculation and its verification of transmission, image capture, image preprocessing, and data organization. The workflow is summarized in Fig. 2.

### 4.1 Greenhouse setup and movable camera platform

**Fig. 3:**
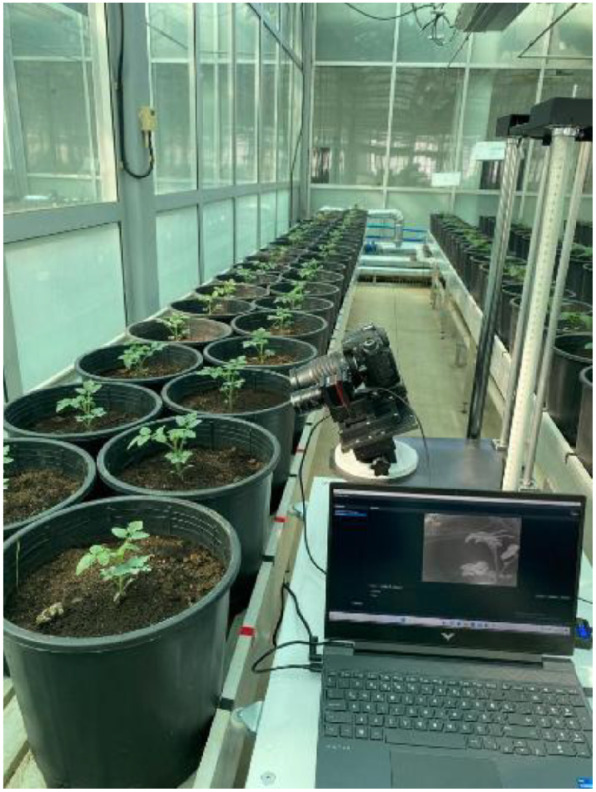
Arrangement of the flowerpots in the greenhouse and movable camera platform used for image acquisition.

A total of 347 plants were used in our study. The distribution of plants between healthy and virus-infected groups is summarized in Table 2. The plant seedlings were transplanted into 17-liter garden flowerpots (31 cm radius, 29 cm height) once they developed 3-4 leaves. The flowerpots were arranged in separate greenhouses, and the placement of flowerpots in one greenhouse is shown in (Fig. 3) Researchers at the Adana Biological Control Research Institution managed plant growth and development through regulated irrigation, precise fertilization, and nutrient management practices. The process also included mechanical virus inoculation and verification of its transmission, complemented by integrated pest and disease management measures to protect the plants from other pathogens and pests.

**Table 2:** Distribution of tomato and pepper plants between healthy and ToBRFV-infected groups used in the study.

Commercial Cultivar	Group	Number of Plants
Tomato I	Healthy	37
	Virus-Infected	45
Tomato II	Healthy	40
	Virus-Infected	45
Pepper I	Healthy	45
	Virus-Infected	45
Pepper II	Healthy	45
	Virus-Infected	45

To capture images with two cameras, a movable camera platform was designed. Both cameras were mounted on an adjustable aperture frame that can be moved horizontally, allowing images to be acquired from the same position. The height of the frame can also be adjusted to obtain an optimal view of the growing plants. The frame also moves in a circular path, and the camera angle can be set in 5° increments using the scale on the frame (Fig. 3).

Two rollers were mounted on the sides of the mobile platform. These rollers remain in contact with the side strips of the table, ensuring that images are captured at a consistent distance. Black and red labels were attached to the side strip of the table to ensure that the platform was positioned consistently for each pot.

### 4.2 Mechanical inoculation of ToBRFV and verification of its transmission

**Fig. 4:**
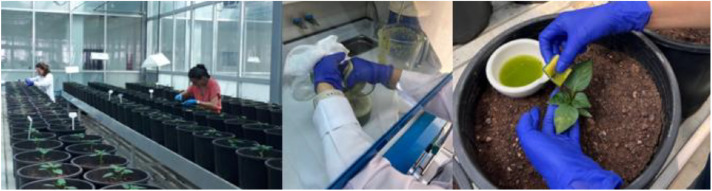
Mechanical inoculation of plants with ToBRFV.

Mechanical inoculation of ToBRFV onto tomato plant leaves and verification of its transmission were conducted by the researchers at the Adana Biological Control Research Institution. For this process, firstly ToBRFV isolate was crushed using a mortar and pestle in 0.01 M phosphate buffer (pH: 7.0) (Na2HPO4 - KH2PO4) containing 0.1% 2-mercaptoethanol at a ratio of 1:5 and then inoculum was prepared by filtering through cheesecloth. The virus isolate was prepared and applied to the cotyledons and first true leaves of tomato and pepper plants using a sponge (Fig. 4). After inoculation, plants was rinsed with tap water, to remove the abrasive and the procedure was repeated on the upper leaves one week after [[11], [12], [13]]. In the experiments, preventive measures were implemented to protect the plants against other diseases and pests.

Transmission was verified using RT-PCR and real-time RT-PCR. Following the second inoculation, fresh leaf tissue was collected on days 7 and 14, and total RNA was extracted using an Norgen brand RNA purification kit. RT-PCR implemented for confirmation of ToBRFV infection resulting the mechanical inoculation.

### 4.3 Data collection

**Fig. 5:**
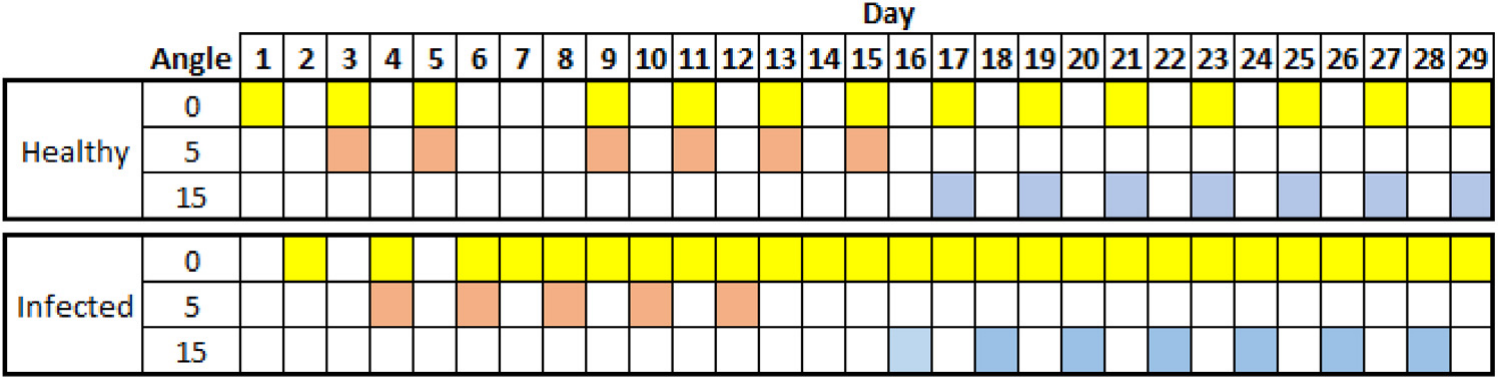
Imaging schedule for healthy and ToBRFV-inoculated tomato plants, showing the time points of image acquisition and the camera angles used during the 29-day experiment.

The experiment was carried out in fully controlled greenhouses at the Adana Biological Control Research Institution between 5 September and 3 October 2023 (29 days). For each plant, images were captured in four modalities: RGB, full-range VNIR (400–1100 nm), VNIR-800 nm, and VNIR-1000 nm. Two cameras were used: Nikon D90 DSLR with AF-S DX Nikkor 18–55 mm f/3.5–5.6G VR lens for RGB imaging, and MT-MIMC-V6418 VNIR camera with Navitar f/0.95–25 mm lens for visible–near-infrared imaging [14]. Narrowband images at 800 nm and 1000 nm were obtained by mounting MidOpt bandpass Infrared (IR) filters on the VNIR lens. DSLR images were acquired with autofocus, while the VNIR camera required manual focusing. Since all images were captured under natural lighting, sunlight reflections occasionally caused overexposure, which was corrected by manually adjusting the VNIR camera’s contrast and brightness. Camera height was adjusted as plants grew to ensure proper framing. The imaging schedule for healthy and virus-infected plants, along with the corresponding camera angles used in the experiment, is provided in Fig. 5. The main experimental conditions and acquisition settings used during data collection are summarized in Table 3. The technical specifications of the RGB and VNIR imaging devices used in this study are summarized in Table 4.

**Table 3:** Description of data collection.

Plants	Tomato and Pepper
Location	Adana Biological Control Research Institution, Türkiye, 37.00° N, 35.32° E
Environment	Fully controlled greenhouses for healthy and ToBRFV infected plants
Experimental period	29 days
Inoculation time	05 September 2023, 11 September 2023 days
Sampling time	05 September – 3 October 2023, Specific daily intervals
Climate	Sunny day
Temperature	∼ 26 − 28 °C in greenhouses
Day light duration	∼ 16 hours
Illumination	Natural light (greenhouse)

**Table 4:** Description of camera devices.

Manufacture	Nikon	Mikro-Tasarım (Türkiye)
Model	D90 DSLR	MT-MIMC V6418 CMOS VNIR Camera
Lens	Nikon AF-S DX Nikkor 18–55 mm	Navitar 25 mm. Midopt bandpass IR filters: 800 nm and 1000 nm.
Aperture	f/3.5	f/0.95
Bit depth	24-bit (RAW)	8-bit (digital video resolution)
Focus/Brightness	Auto	Manual

### 4.4 Preprocessing

The preprocessing pipeline consisted of three main steps: (i) cleaning, (ii) format conversion, and (iii) cropping, (iv) labeling, and (v) data organization. First, a manual data quality analysis was performed by visually inspecting all collected images in four modalities. Images with excessive exposure, strong sunlight reflections, poor lighting, blurring caused by sudden camera movement, duplicate file names, or other quality issues were identified. In such cases, all images of the affected plants from that day were removed to ensure consistency. During the quality analysis, we observed that images from the healthy group captured on Day 7 with the VNIR camera exhibited some of the aforementioned issues. As a result, all images from the healthy group on that day were removed. The number of images for each imaging modality, corresponding to tomato and pepper cultivars, is summarized in Table 5. Across the four imaging modalities, the total number of images for the healthy and virus-infected groups are 16,000 and 24,716, respectively.

**Table 5:** The number of images for one imaging modality in the dataset.

Commercial Cultivar	Number of Images	
	Healthy	Virus-Infected
Tomato I	849	1547
Tomato II	964	1448
Pepper I	1071	1571
Pepper II	1116	1613

**Fig. 6:**
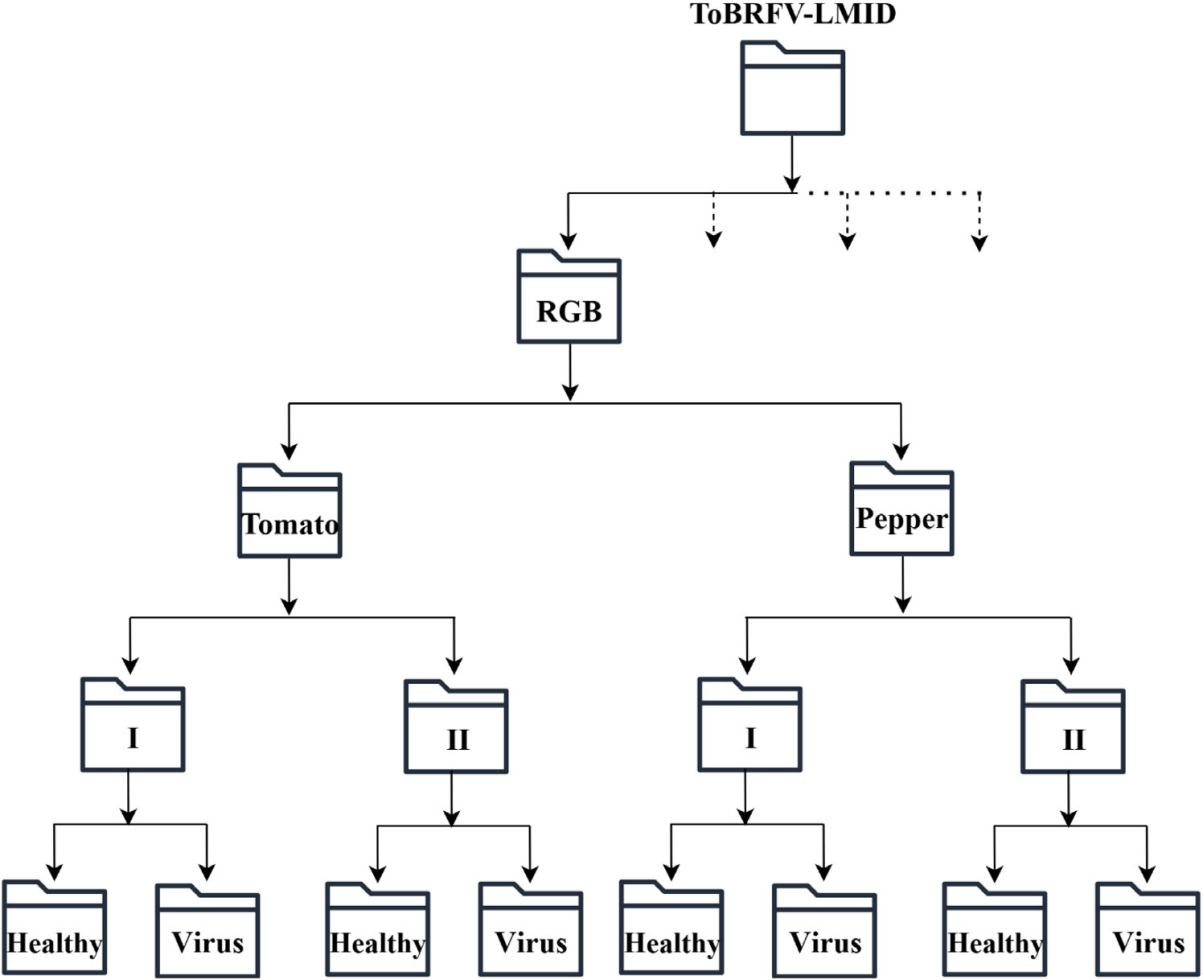
Folder structure.

Second, images acquired with the DSLR camera were originally stored in RAW format. To obtain a standard RGB representation, these images were converted into TIFF format using the DCRAW utility. Third, a rectangular cropping procedure was applied to enclose only the plants in the images. This step was taken to minimize the risk of machine learning models capturing background-related features. The image sizes vary due to the cropping process. Each cropped image was systematically labeled using the format: Virus_Healthy_Day_Camera_Bandpass_IR_Filter_Angle_PotID, with extensions in both TIF and PNG. For example, the file name V_4_VNIR_800nm_5_12.tif indicates that the image belongs to a virus infected plant in pot 12, taken on the fourth day of the experiment using the VNIR camera at 800 nm with a 5° angle. When the VNIR lens was only used without any bandpass IR filter, the corresponding field in the label was left blank. The labeled images were organized into four folders: RGB, VNIR, VNIR_800nm, and VNIR_1000nm. The dataset is distributed as four separate ZIP archives. The folder structure for RGB images is illustrated in Fig. 6.

## Limitations

The dataset was primarily designed to capture morphological responses of tomato and pepper plants to ToBRFV infection. While it includes comprehensive imaging data and verified infection status, it does not provide detailed environmental parameters such as light intensity, humidity, or temperature, nor does it contain agronomic records beyond the validation of ToBRFV transmission. As the experiment was conducted under fully controlled conditions, users should be aware that the dataset focuses only on visual and morphological features of infection, not on broader environmental or agronomic factors. They should also be aware that the four-modality images may be slightly misaligned due to shifts at the aperture and/or the movable camera platform.

## Ethics Statement

The proposed data does not involve human subjects, animal experiments, or data collected from social media platforms.

## CRediT Author Statement

**Rifat Edizkan:** Conceptualization, Methodology, Visualization, Project administration, Writing original draft, Writing–review & editing. **Feyza Yılmaz:** Conceptualization, Investigation, Methodology, Software, Visualization, Writing original draft, Writing–review & editing, Data curation. **Pelin Keleş Öztürk:** Conceptualization, Methodology, Writing–review & editing, Validation, Resources. **Şefika Yavuz:** Methodology, Investigation, Resources. **Ömer Nezih Gerek:** Conceptualization, Methodology, Writing–review & editing. **Hasan Serhan Yavuz:** Conceptualization, Methodology, Writing–review & editing. **Serkan Önder:** Conceptualization, Methodology, Writing–review &editing. **Davut Keleş:** Methodology, Investigation.

## Data Availability

ZENODOToBRFV-Longitudinal Multispectral Image Dataset (Original data)

## References

[1] Cambrón-Crisantos J.M., Rodríguez-Mendoza J., Valencia-Luna J.B., Alcasio-Rangel S., García-Ávila C.D.J., López-Buenfil J.A., Ochoa-Martínez D.L. (2018). Primer reporte de tomato brown rugose fruit virus (ToBRFV) en michoacán, méxico. Rev. Mex. Fitopatol..

[2] EPPO (July 2, 2025). Tobamovirus fructirugosum (tobrfv) – distribution. https://gd.eppo.int/taxon/TOBRFV/distribution.

[3] Salem N., Mansour A., Ciuffo M., Falk B.W., Turina M. (2016). A new tobamovirus infecting tomato crops in jordan. Arch. Virol..

[4] Ministry of Agriculture and Forestry of the Republic of Turkey (2019). Tomato brown rugose fruit tobamovirus (ToBRFV). https://www.tarimorman.gov.tr/GKGM/Belgeler/DB\_Bitki\_Sagligi/Survey/42Tomato\_brown\_rugose\_fruit\_tobamovirus\_(ToBRFV)\_(2019).pdf.

[5] Oladokun J.O., Halabi M.H., Barua P., Nath P.D. (2019). Tomato brown rugose fruit disease: current distribution, knowledge and future prospects. Plant Pathol..

[6] Çelik N., Özalp R., Çelik I. (2024). An open-access tomato brown rugose fruit virus dataset. https://papers.ssrn.com/sol3/papers.cfm?abstract_id=4718071.

[7] Zinger A., Faigenboim A., Gelbart D., Lapidot M. (2025). Creation of a computer vision dataset for tomato brown rugose fruit virus detection. https://www.researchgate.net/publication/391186218_Proof_of_Concept_Creation_of_a_Computer_Vision_Dataset_for_Tomato_Brown_Rugose_Fruit_Virus_Detection.

[8] Wei T., Chen Z., Yu X., Chapman S., Melloy P., Huang Z. (2024). Plantseg: A large-scale in-the-wild dataset for plant disease segmentation. https://arxiv.org/abs/2409.04038.

[9] Singh D., Jain N., Jain P., Kayal P., Kumawat S., Batra N. (2019). Plantdoc: A dataset for visual plant disease detection. https://arxiv.org/abs/1911.10317.

[10] Luijk G. (2007). Dcraw tutorial. https://www.guillermoluijk.com/tutorial/dcraw/index_en.htm.

[11] Deom C.M., Quan S., He X.Z. (1997). Replicase proteins as determinants of phloem-dependent long-distance movement of tobamoviruses in tobacco. Protoplasma.

[12] Çelik N., Özalp R., Çelik I. (2010). Bazı biber hat ve Çeşitlerinin tobacco mosaic tobamovirus (tmv)’e dayanıklılığının mekanik İnokulasyon ve elisa testleri İle belirlenmesi. Batı Akdeniz Tarımsal Araştırma Enstitüsü Dergisi.

[13] Ozturk P.K., Argun D., Baloglu S., Keles D. (2020). Effect of tobacco etch virus (tev) on yield and quality of red pepper in turkey. Acta Sci. Pol. Hortorum Cultus.

[14] Mikro-Tasarım (2025). MT-MIMC-V6418. https://www.mikro-tasarim.com.tr/solution/mt-mimc-v6418/.

